# Special Notice

**Published:** 1880-12-20

**Authors:** 


### EDITORIALS AND MISCELLANEOUS.

### SPECIAL NOTICE.

*Renewals.*—All our subscribers who do not notify us to the contrary, on or by the 15th of January next, will be considered as having renewed their subscriptions and will be entered on the list for 1881.

We hope, friends, that you will not only allow us so to enter your names, but that each one will send us at least one new subscriber. If so, will allow you club rates—$1.50 each or $3.00 for yourself and a new subscriber. In this case the cash should accompany the order.

